# First Principles Modeling of Nonlinear Incidence Rates in Seasonal Epidemics

**DOI:** 10.1371/journal.pcbi.1001079

**Published:** 2011-02-17

**Authors:** José M. Ponciano, Marcos A. Capistrán

**Affiliations:** 1 Department of Biology, University of Florida, Gainesville, Florida, United States of America; 2 Department of Mathematics, Centro de Investigación en Matemáticas (CIMAT), Guanajuato, México; University of Michigan and Howard Hughes Medical Institute, United States of America

## Abstract

In this paper we used a general stochastic processes framework to derive from first principles the incidence rate function that characterizes epidemic models. We investigate a particular case, the Liu-Hethcote-van den Driessche's (LHD) incidence rate function, which results from modeling the number of successful transmission encounters as a pure birth process. This derivation also takes into account heterogeneity in the population with regard to the per individual transmission probability. We adjusted a deterministic SIRS model with both the classical and the LHD incidence rate functions to time series of the number of children infected with syncytial respiratory virus in Banjul, Gambia and Turku, Finland. We also adjusted a deterministic SEIR model with both incidence rate functions to the famous measles data sets from the UK cities of London and Birmingham. Two lines of evidence supported our conclusion that the model with the LHD incidence rate may very well be a better description of the seasonal epidemic processes studied here. First, our model was repeatedly selected as best according to two different information criteria and two different likelihood formulations. The second line of evidence is qualitative in nature: contrary to what the SIRS model with classical incidence rate predicts, the solution of the deterministic SIRS model with LHD incidence rate will reach either the disease free equilibrium or the endemic equilibrium depending on the initial conditions. These findings along with computer intensive simulations of the models' Poincaré map with environmental stochasticity contributed to attain a clear separation of the roles of the environmental forcing and the mechanics of the disease transmission in shaping seasonal epidemics dynamics.

## Author Summary

Nonlinearity in the infection incidence is one of the main components that shape seasonal epidemics. Here, we revisit classical incidence and propose a first principles derivation of the infection incidence rate. A qualitative analysis of the SIRS model with both the classical and the proposed incidence rate showed that the new model is physically more meaningful. We conducted a statistical analysis confronting the SIRS and SEIR models formulated using both incidence rate functions with four data sets of seasonal childhood epidemics. Two data sets were hospital records of cases of syncytial respiratory virus (RSV). The other two data sets were taken from the well-known UK measles epidemics database. We found that seasonal epidemics is better explained using our incidence rate model embedded in a Poisson sampling process. The results presented here are not by any means an exhaustive exploration of the interplay between nonlinear dynamics and stochasticity. Our results may be viewed as the starting point of multiple research avenues. Three such research topics could be: the first-principles derivation of non-linear incidence rate functions, the role of bistability and demographic stochasticity for disease persistence and the simulation of environmental and demographic stochasticity in the Poincaré map.

## Introduction

A plethora of deterministic epidemic models involving susceptible 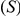, infected 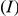 and recovered 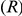 individuals have been proposed [1], [2], carefully analyzed [3]–[8] and confronted with data sets in the biomathematics and ecology literatures [9]–[12]. A well defined topic within this mathematical ecology research area is the study of 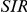-type models with seasonal forcing [13]–[16]. These models have proved to be useful for understanding the observed patterns and the natural processes behind human and non-human epidemics [17]–[21]. Here, we restrict our attention to the 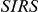 and 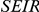 models in which we introduce seasonal forcing while varying the structural form of the incidence rates. Two hypotheses pertaining the RSV and the measles transmission mechanisms were modeled with two simple functional forms of the incidence rates. We show that in doing so, we are able to attain a clear separation of the roles of the environmental forcing and the mechanics of the disease transmission in shaping the epidemics dynamics.

The construction of deterministic incidence rates functions is a critical building block of epidemiological modeling. In a seminal paper, Hethcote [1] showed that because there are many choices for the form of the incidence, demographic structure and the epidemiological-demographic interactions, there really is a plethora of incidence rate functional forms to choose from. Not surprisingly, the biomathematics literature abound in qualitative mathematical analyses of many of these functional forms [22]–[26]. However, biological first principles derivations of incidence rate functional forms are not too common. As we show in this study, using such first principles derivations greatly enrich the reaches of the practice of confronting models with data while testing biological hypotheses. Thus, despite the big amount of available functional incidence rates forms [1], we believe that the set of models chosen to be confronted with data should be restricted to those forms derivable from first principles. To illustrate this argument, in this study we first show that a simple probabilistic setting wherein infectious encounters are modeled with a pure birth stochastic process leads to a general nonlinear incidence form proposed previously by Liu [24] and later analyzed by Hethcote and Van Den Driessche [23] (hereafter we refer to the Liu, Hethcote and Van Den Driessche incidence rate as the LHD incidence rate). The LHD incidence rate leads to models with qualitatively different dynamics compared with the ones obtained using the classical incidence rate.

In the SIRS model with either incidence rate and seasonal forcing, 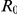 becomes a periodic function of time and the trajectory 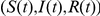 “pursuits” a moving target thus giving rise to limit cycles. That moving target is the former endemic equilibrium that bounces back and forth between two points. In either model, the target switches between that moving point and the disease free equilibrium when 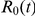 crosses 1, giving rise to a period doubling bifurcation. In the SIRS model with classical incidence rate this mechanism does not depend on the initial conditions. In this work we show that the disease free equilibrium (DFE) is unconditionally an attractor in the SIRS model with LHD incidence rate. This leads to a scenario where two regions of attraction can coexist. The trajectory 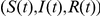 will either reach the disease free equilibrium or have periodic solutions depending on the initial conditions. Furthermore, after carrying a formal model selection we show that the SIRS model with LHD incidence rate leads to a significant fit improvement over the classical SIRS model with the same seasonal forcing. Finally, we compared the applicability and generality of the classical and LHD incidence rates functions by fitting them to two measles time series data sets. Using the later function leads to a vast improvement of model fit in both cases. Since we were fitting a deterministic SEIR model, we chose to use the data from the two largest cities in the measles data set (London and Birmingham, see http://www.zoo.cam.ac.uk/zoostaff/grenfell/measles.htm), where the effects of demographic stochasticity are expected to be less influential in the dynamics of the epidemics [10].

Varying the form of the contact rate function while including environmental stochasticity in the SIRS and SEIR models leads to a better understanding of the dynamics of an infectious disease transmission. Depending on the model and contact rate, the disease free equilibrium (DFE) is either a saddle point or an attractor. In the first case, if a trajectory located originally in the basin of attraction of the endemic equilibrium (EE) basin of attraction is perturbed with environmental noise, it may transiently visit the DFE stable submanifold and then return to the EE basin of attraction. If however the DFE and the EE coexist as stable equilibria, a trajectory initially at the EE basin of attraction may end up in the DFE basin of attraction. The interaction between stochasticity and the different contact rate models was studied using computer intensive simulations of the Poincaré map [27].

## Model

### SIRS dynamics

The classical 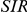 model has been extensively studied in order to predict and understand various disease dynamics behaviors, as well as their spread and persistence [28]. For many infectious diseases, the pool of susceptible individuals is replenished due to the waning of immunity [17], [18]. To account for the lost of immunity, the classical susceptible 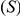, infected 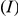 and recovered 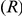 model is adjusted by allowing a fraction of the recovered individuals 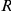 to move back into the susceptible pool 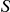 at a rate 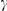. This susceptible, infected, recovered and susceptible 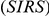 model is expressed as
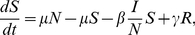
(1)

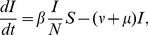
(2)

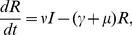
(3)where 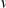 is the rate of loss of infectiousness and the total population size remains constant (*i.e.* 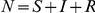). The constant 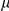 represents both, the birth and mortality rates. Assuming that birth and mortality rates are equal is justified on the grounds that the annual infection rate is considerably higher than the population growth. The constant 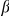 is the contact rate, the average number of individuals with whom one infected individual makes sufficient contact to pass on the infection [29]. The fraction 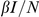 represents the average number of infections per susceptible individual and hence 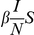 represents the expected number of infections when 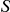 susceptible individuals are available [5]. Note that the above definition of 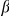 as a per individual constant leads to a consistency of the units within each of the model equations and assumes homogeneous mixing. In the following sections we will discuss different ways to model the incidence rate.

### SEIR dynamics

The equations for the classic SEIR (Susceptible-Exposed-Infectious-Recovered) model are as follows [30]:
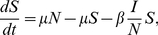
(4)

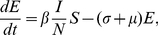
(5)

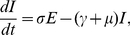
(6)

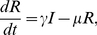
(7)where 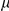 represents both, the birth and mortality rates per capita. The mean latent and infectious periods of the disease are 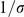 and 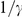. As written, the SEIR model has a stable endemic equilibrium provided 
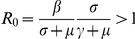
. Further biological realism to model recurrent epidemics can be incorporated to both this SEIR model and the SIRS model above by assuming that the transmission rate varies seasonally. Indeed, Earn et al [30] study the range of the dynamical behavior of the SEIR model with seasonality and find it useful for explaining the measles numerous transitions between regular cycles and irregular, possibly chaotic epidemics. Also, Alonso et al. [31] show that noise amplification provides a possible explanation for qualitative changes from regular to irregular oscillations of lower amplitude. In this paper, we follow the suggestion made by Hethcote [1] and couple Liu, Hethcote and Van Den Driessche's incidence rate with seasonal forcing in both the SIRS and SEIR models.

### Seasonal forcing

To incorporate the claim that epidemics of recurrent infections is driven by seasonality, it is customary to depart from the standard incidence rate 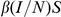 by assuming that the average number of incidences sufficient for transmission per infected individual 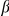, is a periodic or quasi-periodic function of time (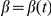). Often, the incidence rate is assumed to have a sinusoidal form of the type
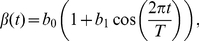
(8)where 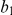 stands for the strength of the seasonality and 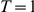 year. Various authors have shown that such a generic description of the seasonal variation in transmission rates is not as revealing as a detailed description of the actual processes underlying the seasonal drivers of transmission through mechanistic seasonal forcing functions [11], [18], [30], [32], [33]. However, as we show in the results section, in some cases this sinusoidal function may unequivocally represent a linear transformation of a weather covariate. Although other authors have used a more flexible Haar step function for the seasonal forcing (*e.g.* [30]), we restrict ourselves to the incorporation of the sinusoidal form above (eq. 8) as the seasonal forcing. This has the advantage of ease of interpretation and qualitative analysis. In any case, the main purpose of incorporating the forcing is to explore the main qualitative characteristics of coupling the seasonally varying disease transmission and different incidence rate functional forms.

### First principles modeling of incidence rates

Brauer [34] generalizes the incidence rate definition in the following way: if the average member of the population makes 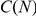 contacts in one unit of time with 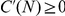, and if 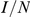 is the probability of choosing one infected individual from the population at random, then 
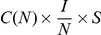
 is the rate of new infections per unit of time. The mass-action incidence rate model 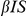 is recovered using 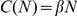 and the classic incidence rate is recovered by picking 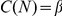. A general incidence rate function was proposed by Hethcote and van den Driessche [23]:
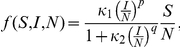
where 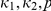 and 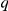 are constants. Consider the special case where 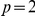 and 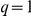. Using Brauer's generalization and idea, Hethcote and van den Driessche's model is recovered using the function 
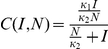
. Then, the incidence rate function becomes
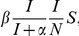
where 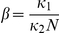 and 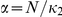. Although the mathematical properties of the general function are known in general [23], [35], [36] a mechanistic, first principles derivation of it is still lacking. Such a derivation can be obtained using a probabilistic reasoning analogous to the argument used by [37] to model the Allee effect through stochastic mating encounters:

Through physical movement or any other means of dispersion, an infected individual will have contact with a given number of susceptible individuals in the population. The potential to effectively disperse the disease (virus) could be thought of as being proportional to that number of susceptibles with whom the infected individual makes contact: indeed, the more contact the infected individual has with susceptibles, the more likely he is to effectively transmit the disease. It then follows that the magnitude of the realized disease dispersion could be measured for example, in terms of the dispersion ability (i.e. vagility) of the infected individual. Accordingly, every infected individual will be expected to realize a certain virus (or micro-parasite) dispersion potential. Let the realized disease dispersion made by one infected individual be denoted by 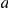. Then, the number of successful transmission encounters per infectious individual can be modeled with a random variable 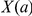. By writing 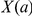, we are stressing the fact that the infection process is a function of the magnitude of the realized dispersion. Furthermore, we assume that the probability that an infected individual encounters and infects a susceptible individual given a realized change in dispersion 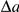 is proportional to the previous number of successful infection encounters times a function 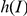 of the number (or density) of the infected individuals in the population. Often [7], a non-linear function 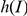 is chosen to account for factors such as crowding of infected individuals, multiple pathways to infection, stage of infection and its severity or protective measures taken by susceptible individuals. These assumptions allow us to specify a new infection event as the conditional probability
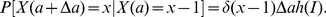
(9)where 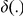 is a non-negative function such that 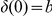 is a constant. Towards the end of this section we discuss possible functional forms for 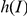. We remark that if 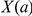 counts the number of successful transmission encounters of an infected individual that recently invaded a population consisting only of susceptible individuals, then the expected value of 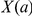 is in fact equal to the mean number of secondary infections 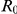 in the context of the SIRS model. If the SEIR model dynamics is in place, then, when there is only one infected individual in the population, 
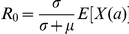
. Assuming that the probability that more than one successful infectious encounter occurs after an extra dispersion amount 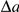 is negligible, then 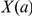 can be modeled using a simple homogeneous birth process where the quantity being born is the number of successful virus transmission encounters. The probabilistic law of this stochastic process is completely defined by the terms 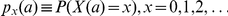. To solve for these terms, first note that according to eq. (9)
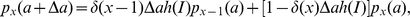
which leads to
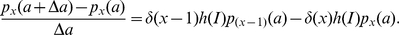
In the limit when 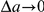, the above equation leads in turn to the following system of differential equations:
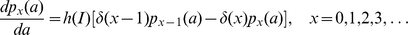
Then, it is well known [38] that solving this system of equations leads to
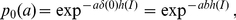
(10)

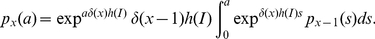
(11)Furthermore, approximating 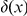 using a Taylor series expansion around 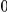 leads to specific quantitative definitions of the stochastic process 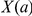. For example, if 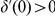 or if 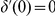, the one-step transition probability mass function (pmf) of 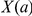 adopts the negative binomial and Poisson forms respectively [37]. The Negative Binomial transition pmf would bring into the picture over-dispersion (higher variance to mean ratio) as a key qualitative property of the moments of the pure birth process describing the evolution of the number of successful transmission encounters. In any case however, the probability that one infected individual successfully passes on the infection is
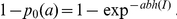
This expression is readily interpretable: for a fixed value of 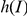, the probability of successfully passing on the infection converges to 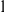 as the product 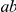 grows large. Therefore, in this expression we are recovering the model property that the probability of successfully passing on the infection increases with the realized disease dispersion effort 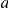. Each individual's realized dispersion is in turn related to the individual's ‘effort’ to transmit the infection. In a given population, the magnitude of the realized disease dispersion for each infected individual can be expected to vary widely. Accounting for this demographic source of heterogeneity could be achieved by assuming that each individual's dispersion ability is drawn from a given probability distribution. That is, we would be modeling the variation in disease dispersion per infected individual with a random variable 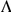 whose pdf 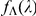 has support on 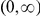. Without loss of generality, here we model randomness in the product 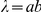 instead of just in the realized disease dispersion 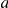. Then, the probability that an infected individual chosen at random from the population realizes more than one successful secondary infection is found by averaging 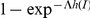 over all the possible realizations of 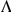. That is,
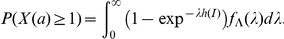
A suitable probabilistic model for 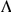 with empirical and theoretical support can be difficult to find (see for instance the models in [39]). A flexible positive, continuous distribution such as the gamma distribution could therefore be used. Here, we assume that the magnitude of the disease dispersion brought about by an infected individual is distributed according to a special case of the gamma pdf, the exponential distribution. Accordingly, letting 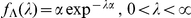 we get that the probability of successfully transmitting the infection is
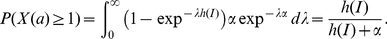
(12)As mentioned before, various biological hypotheses pertaining the behavior of the transmission as a function of the abundance of infected individuals have been advanced to justify various functional forms of 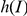. Suitable candidates for 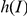 should satisfy the conditions
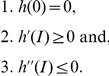
These conditions guarantee the basic requirement that the probability of a new infective encounter (eq. 9) is null in the absence of infected individuals and that the overall chance that a new infection occurs increases proportionally with 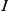 when 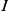 is small. Furthermore, if 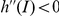 such proportionality decreases in magnitude as 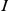 grows large (that is, 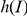 is concave down). Consider the following two functional forms:

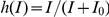
, where 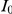 is a constant. This model whose second derivative is negative, was first proposed by Capasso and Serio [22] to account for saturation of infected individuals. Substituting this functional form in eq. (12) we get that
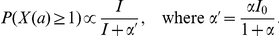
Note that here, the biological hypothesis of saturation is translated into a model using a phenomenological argument: the functional form of the model mimics a hypothesized pattern instead of modeling the biological process generating the pattern.

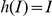
. This function is the simplest way to satisfy the three conditions above without introducing an extra parameter and/or a phenomenological modeling approach. However simple, when substituted in eq. (12) we still recover the same functional form for the probability of at least one successful transmission encounter, that is
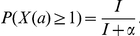
The exponential distribution parameter 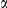 takes here an important meaning: it is the density of infected individuals at which the probability of successfully transmitting the infection is 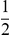. It also follows from this argument that the incidence rate function can be modeled as a constant times the probability of picking an infected individual at random in the population times the probability that an infected individual successfully passes on the infection times the total number of available susceptibles in the population. That is,
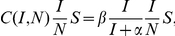
which is Liu's and Hethcote and van den Driessche's model with 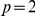 and 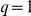. This incidence rate function explicitly states that the transmission rate is proportional to the number of available susceptible individuals 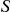 and that the constant of proportionality is a function of the number of infected individuals. Also, we would like to stress that, by taking into account the per-individual variability in dispersion abilities, this formulation of the incidence rate function goes from individual-based processes to population-wide patterns in disease transmission. The effect of different hypotheses pertaining individual-based contagion processes into the population-level disease transmission processes could be tested by proposing different -biologically meaningful- probability distributions of the infected individuals potential to disperse the disease.

Many other functional forms 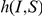 for the incidence rate could be derived using the above arguments. If for instance other heavy-tailed distributions are used instead of the exponential distribution, other incidence rate functional forms will arise and this could certainly be the topic of further research. However, in this work we limit ourselves to the exploration of the reaches of using the LHD model because it explicitly incorporates heterogeneity in transmission potential, because of its bi-stability properties (see “qualitative analysis of the SIRS models” section) and to formally test if it arises as a better explanation for bi-annual epidemic patterns using data from different localities and diseases. Thus, from this point on, in this work we will only consider the LHD incidence rate function and the classical incidence rate 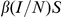. In his seminal paper, Hethcote [1] also mentions that the LHD general incidence rate function could be eventually coupled with any seasonal forcing function. Motivated by this comment, in the results section we explore the reaches of doing so.

### Materials and methods

#### RSV data analysis

**Figure 1:**
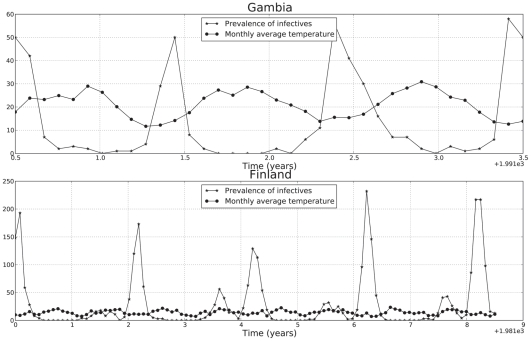
Observed time series of infected individuals in Gambia and Finland. Plotted are the monthly number of reported syncytial virus cases in two cities: Banjul in Gambia (from October 1991 to September 1994) and Turku in Finland (from October 1981 to March 1990). Plotted also is the mean monthly temperature range for both localities, for the same time spans.

The parameters for the SIRS model with two different incidence rate functions were estimated *via* maximum likelihood [40] using time series data from two localities in Gambia and in Finland (Figure 1, data kindly provided by Prof. A. Weber, see also [9]). For each geographical locality, the data consists of the pairs 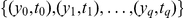, where 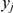 denote the reported number of cases (*i.e.* incidence) at time 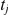, for a total of 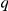 time steps. In both localities the size of the time step is a month. Because the data of infected individuals consists of counts, a natural and simple statistical sampling model is the Poisson distribution [17], [41]–[43]. Heterogeneity in sampling effort or other sources of heterogeneity in the sampling scheme could be accounted for using the negative binomial distribution, but we consider that the Poisson model is a fairly robust description of the situation faced with this data sets (see [42], sub-section “Observation error models” in the “Discussion”). Therefore, we assumed that the observations 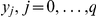 are independent realizations of a Poisson distribution 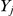 whose mean changes according to the deterministic model predictions. Let 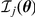 be the predicted number of new cases between times 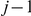 and 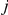 by a SIRS model evaluated at the vector of parameters 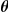, that is:
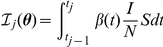
for the classic SIRS model and
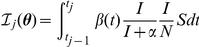
for the LHD SIRS model. For the SEIR model with either incidence rate functions, the model predicted number of new cases was computed as 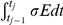. To carry the numerical integrations, we used Romberg's method (see [44] and links to programs in Supporting Information). We assumed that the first observation 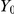 arose from the process at stationarity, that is, once the limit cycles predicted by both models had been reached. The biological reasoning behind this assumption is the fact that the infectious process of interest is a well-established disease that has evolved a stable dynamics and is under the influence of a stationary climatic process. The above assumptions allow us to adopt the Poisson sampling model
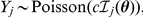
where the constant of proportionality 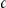 involves the infected individuals' detection probability (see [45] p. 10).

Assuming that the observations are independent between them, the joint distribution of the observed infected individual abundances 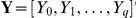 is a good approximation to the likelihood function 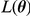 [46], which would simply be defined by the product of the individual pdf's of the observations:
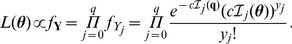
(13)The maximum likelihood (ML) parameter estimates for 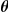, denoted 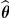 are the values of 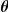 that jointly maximize 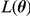. That is, the ML estimates are the solution to
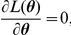
which is equivalent to solving 
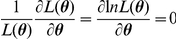
. Accordingly, the parameter values that minimized the negative log-likelihood
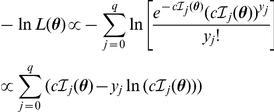
were taken to be the ML parameter estimates. The minimization was carried using the L-BFGS algorithm of Zhu *et al* [47]. The computer code written in Python 2.6.1 used in this work can be found as a supplement under the title “Dataset S1”.

Additional information about the weather was also incorporated in the parameter estimation process. In particular, the mean monthly temperature range data available at 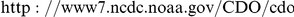, meteorologic stations 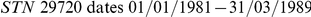 and 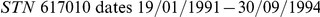 were used as weather covariates to find the ML estimates of the models parameters. This weather variable has a strikingly strong sinusoidal pattern that has the same periodicity than the time series of infected individuals. Also, as shown in Figure 1 the mean monthly temperature range (hereafter simply referred to as “the weather covariate”) and the time series of infected individuals appear to be exactly out of phase: a lower mean monthly temperature range is accompanied by a high reported number of infected cases for the same month. Therefore, to include the weather data in the parameter estimation and modeling processes, we assume that the cosine function (8) denotes the effect of the mean monthly temperature range in the number of infected individuals for the same months. The data to be used for parameter estimation when both, the weather and the weather effects are modeled is composed of the triplets 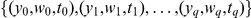, where 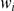 denotes the *observed* mean monthly temperature range for month 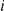. Denote with 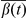 the mean of the cosine incidence rate function (8) and with 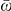 the average of the mean monthly temperature range stationary time series. We assume that the incidence rate 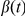 can be modeled with a deterministic linear function of the true weather covariate 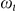:
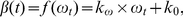
(14)where 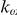 is a factor transforming temperature in incidence rates and 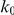 is a reference incidence rate at zero temperature. Solving for 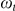 in the above eq. (14) yields
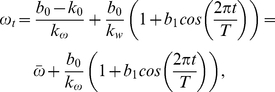
(15)where the RHS is derived by noting that by construction, the independent term 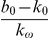 is the average monthly temperature 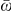. Also, we remark that using the empirical assumption that incidence rates and temperature are exactly out of phase implies that 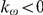.

To connect the time series of observed weather values 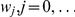, to the model above, we adopt a Normal statistical sampling model. In particular, we assume that these observations are Normal deviates with mean given by 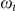 (eq. 15) and constant variance 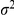. Let 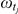 denote the weather model prediction from eq.15 corresponding to the 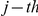 weather observation 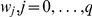. The negative log-likelihood function derived from such statistical sampling model then becomes the score function that is minimized using a numerical algorithm. The likelihood function for the weather data is
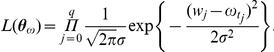
Maximizing this likelihood function to find the ML parameter estimates for the vector 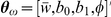 is equivalent to minimize the sum of squares
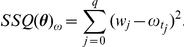
The ML estimate of 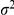 is found in turn by plugging the ML estimates of 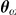 in the likelihood function and solving
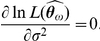
Accordingly, we find that
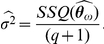
More complex stochastic continuous models that not only model the sampling error (under and over reporting for instance) but also the stochasticity inherent to the weather process will be treated in a future paper. Because information about 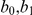 and 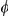 is also conveyed by the time series of infected cases, we maximized the joint likelihood of the time series of infected cases and of the weather data which, from independent sampling is taken to be the product of the individual likelihoods 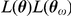. We maximized this likelihood which amounted to jointly minimize the sums of squares 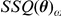 and the negative log-likelihood 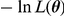 (see eq. 13). The results of the parameter estimation process with and without covariates are reported in the results section.

Finally, previous information about the value of the model parameters 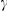, 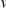 and 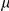 was available in Weber et al. [9]. We fixed these parameters at the values 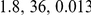 for Finland and 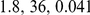 for Gambia. Also, the total population sizes was taken to be 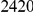 in Finland and 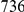 in Gambia. This population sizes are the scaling factors reported in the unit based model of Weber et al. [9]. Once the ML estimates for the two SIRS models and the two localities were found, we proceeded to carry a model selection process using Akaike's information criterion [48]. This procedure allowed us to select amongst the two models at hand (the SIRS model with classical and LHD incidence rate functions) which one appeared to represent a better explanation of the epidemic patterns seen in the time series data. The use of AIC for model selection has a strong theoretical rooting in information theory. For a given data set, the AIC gives an estimate of the expected, relative, directed distance between the fitted models and the unknown true mechanism that generated the data [49]. Thus, the decision rule for model selection is to choose the model with the lowest AIC. Let 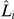 denote the likelihood function for model 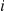 evaluated at the ML estimates and let 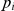 denote the number of parameters used by model 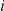. Then, the AIC statistic of model 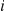 is simply:
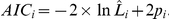
Often, model selection exercises are carried using two or more information criteria. Here, we relied on AIC and on the Bayesian information Criterion (BIC) [50] to simultaneously assess the quality of each model to explain the data at hand. We note that Schwarz [50] showed for a large lass of models that if the true model is among the suite of competing explanations, then the BIC will choose the true model in the limit, as sample size increases, with probability approaching 1 (that is, the BIC is statistically consistent if the true model is in the candidate pool). In real situations, the BIC will select the model in the pool that best approximates the true model. The BIC is calculated with:
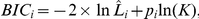
where 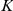 is the total number of data points used in the parameter estimation process. When we used the weather time series besides the time series of infected individuals for parameter estimation we took 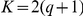. For the models fitted using the simple Poisson likelihood we used 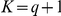. The resulting AIC and BIC values for each model and each locality is shown in the results section. A disagreement between the two statistics would indicate that there is not enough evidence in the data to support the best model, and a decision would have to be taken after investigating the type I error rates of each model using extensive simulations.

#### Measles data analysis

Two time-series from the UK data set (http://www.zoo.cam.ac.uk/zoostaff/grenfell/measles.htm) were chosen: the data for London and Birmingham. According to previous research efforts [10], these UK cities have a population size well above the critical community size, the effects of demographic stochasticity are not expected to be large and the disease was endemic from 1944 to 1966. Further, it has been established that measles in the UK reveals a well defined biennial pattern of major and minor epidemics after the baby boom of 1947 and before the national immunization program started in 1968 [51]. We estimated the parameters of the SEIR model with two different incidence rates with data from London and Birmingham from 1950 to 1959. While analyzing these measles data, other authors have included as seasonal forcing the effect of school terms by means of a term-time forcing function [30], [52]. Although we are aware that this approach leads to more realistic predictions, we constrain ourselves to a simple sinusoidal function since it constitutes a low dimensional approximation amenable to bifurcation analysis.

For each city, the data consists of pairs 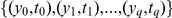, where 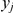 denote the number of reported cases at time 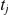, for a total of 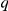 time steps. In both localities the size of the time step is two weeks. To connect the time series data with the SEIR model we used the same approach as with the RSV data set: we assumed that the true infectious process is deterministic and that the observed deviations from the model predictions were due to Poisson sampling error. The minimization of the negative log-likelihood function eq. (13) was again carried using the L-BFGS algorithm. We note that not all the model parameters were estimated. The values for the mean latent and infectious periods were taken to be 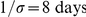 and 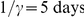 respectively [30].

#### Assessing the effects of environmental stochasticity

A common way to investigate the range of possible dynamic behaviors exhibited by a model is by means of bifurcation diagrams. Kuznetsov [53] and Earn *et al* [30] for instance, illustrate how varying the value of the seasonality and/or the mean contact rate gives rise to saddle-node and period doubling bifurcations. A trajectory that switches between multiple basins of attraction can result from the interaction between stochasticity and complex deterministic dynamics [54]. To assess the effects of environmental stochasticity in the SIRS and SEIR models' dynamics we simulated stochastic dynamics from the associated Poincaré map in the following way [27]:

Consider the discrete map that results from recording the same day every year the solution of the continuous SEIR or SIRS models. Denote this discrete map by 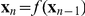, where 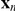 is the vector denoting the recorded solution at year 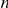. The discrete map was perturbed with environmental noise by multiplying 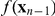 by 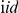 normal random variables 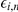, where 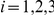 for the SIRS model and 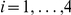 for the SEIR model. With such a perturbation, the growth rate 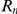 of the discrete map becomes
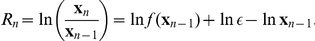
It is well known that a discrete map with environmental and demographic stochasticity is characterized by a growth rate whose variance is 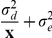, where 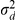 and 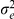 are constants. The signature of environmental noise is that its variance is independent of the size of the state variables [54]. In this case, including the environmental noise according to [27] results in a perturbation in the growth rate 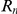 with mean 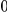 and a variance approximately equal to 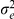.

## Results

### Parameter estimation and model selection

**Figure 2:**
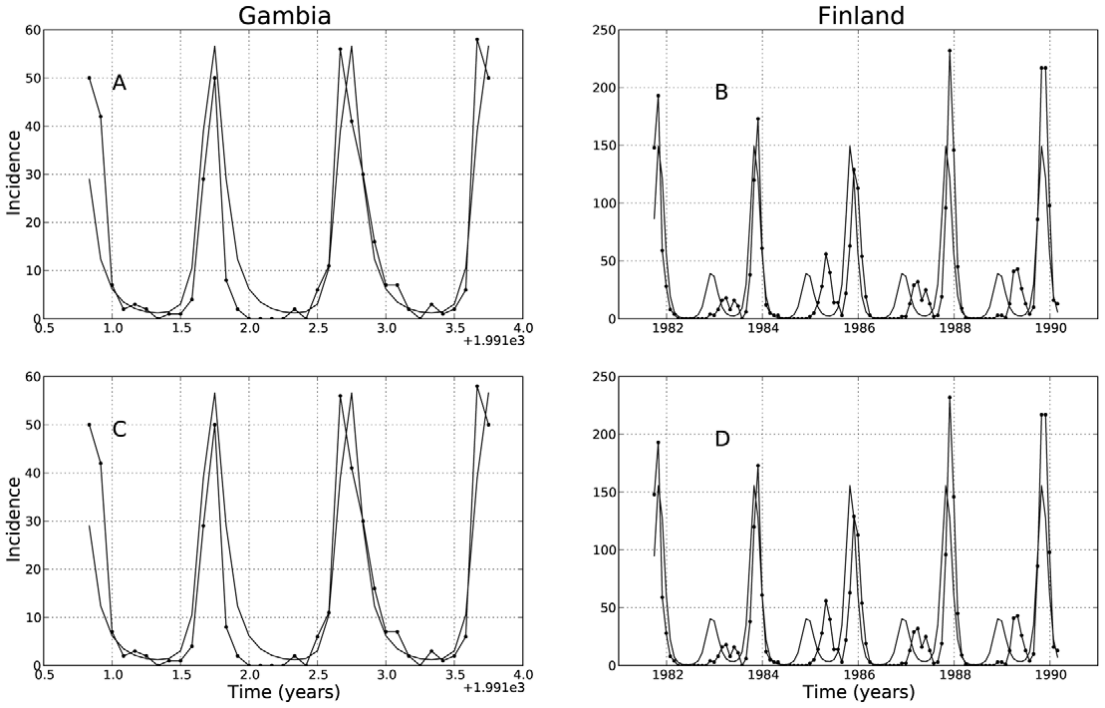
Predicted vs. observed time series of infected individuals. Using the ML estimates in Table 1, the predicted infected dynamics of the classical SIRS model was compared against number of infected individuals reported in Gambia and in Finland. Panels A and B show the predictions for the Classical SIRS model and panels C and D show the predictions for the LHD SIRS model.

The two different SIRS models were fitted to time-course data of reported cases of syncytial virus infections. The data come from Gambia and Finland (Figure 1). Two ML formulations were used. The first one consisted of a Poisson likelihood that only required the available observed counts of infected individuals (eq. 13). The second formulation consisted of the joint likelihood of the counts and of the observed weather covariate and thus used information present on the time series of reported cases and on the corresponding time series of mean monthly temperature range for both locations. The ML estimates according to the first formulation for each model and data set combination are displayed in Table 1. Both information criteria used indicate that for Finland, the best model was the SIRS model with LHD incidence rate function. For Gambia, both information criteria for the SIRS model with classic incidence rate function are lower by three points approximately. This implies that given the data and the two information criteria ways of penalizing the likelihood score, both models are nearly indistinguishable for any practical purpose [49]. In Gambia, the extra parameter introduced by the LHD model is penalized: given the data set at hand, incorporating one extra parameter does not lead to a clear improvement In Figure 2 we plotted the model predicted number of infected individuals versus the observed values for the classical and the LHD SIRS model respectively. Note that, even though the best model is deterministic, the dynamics displayed by the data (small epidemics followed by a big epidemic peak) is very well recapitulated by the predicted solutions.

**Table 1:** RSV-SIRS model parameter estimates and model selection using a Poisson sampling model.

Finland									
Model	P	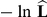	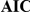	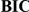	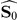	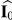	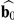	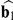	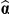
Classic	3	−1647.400	3300.8000	3308.9290	2.1948e+03	8.6808e+01	4.2847e+01	2.9136e−01	NA
LHD	4	−1589.048	3186.0950	3196.9330	2.1858e+03	9.4965e+01	4.2878e+01 (±4.5816E−11)	2.7076e−01 (±3.2131E−10)	5.8830e−03

Maximum likelihood (ML) parameter estimates for both models and two time series of the number of reported syncytial virus cases in two different localities: Gambia and Finland. The statistical model for the observation error is the Poisson distribution. The letter 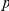 denotes the number of model parameters in each case. 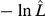 denotes the value negative log-likelihood function evaluated at the ML estimates. The AIC and BIC scores for each model vs. data set combination are also reported. The model selection decision rule is to pick the model with lowest information criterion value. Accordingly, the LHD model seems to be the best choice in Finland whereas the Classical model seems to be a sufficient explanation for the observed time series patterns in Gambia. Confidence intervals for 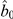 and 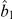 are shown in parentheses for the best model for each locality.

The results of the second ML formulation are qualitatively identical to the results with the Poisson likelihood (see table in the Text S1). For Finland, the BIC statistic for the classical model was 10376.2000 and for the LHD model 9893.5780. For Gambia, the BIC for the classical model was 729.1133 whereas the LHD model had a BIC of 733.2750. Hence, here again, for Finland the LHD is the best model whereas for Gambia, the classic model is better. Because the BIC can be used only to compare models for which the numerical values of the dependent variable are identical for all estimates being compared, it cannot be used to select between the two ML formulations. Indeed, in the second likelihood formulation the data fitted consist not only of the time series of infected counts but also of the monthly temperature range, thus it uses twice as much data for parameter estimation. Zeng et al [55] mention that an indication of which likelihood formulation is better can be obtained by comparing the *per datum* BIC score. Take for instance the BIC for the LHD model for Finland, 9893.5780. Dividing that BIC by the total number of data points used (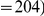, we get a *per datum* BIC of 48.4979. Now, the BIC for the LHD model for the Poisson likelihood formulation is (Table 1) 3196.9330. Dividing that number by the number of data points used (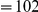) we get 31.34248. Thus, the Poisson likelihood formulation yields a better *per datum* BIC for Finland. For Gambia, the Poisson likelihood formulation seems to be better than the Poisson-Normal sampling model: for the classic model with Poisson likelihood this statistic is 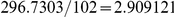, whereas for the classic model with Poisson-Normal likelihood it is 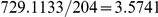.

**Figure 3:**
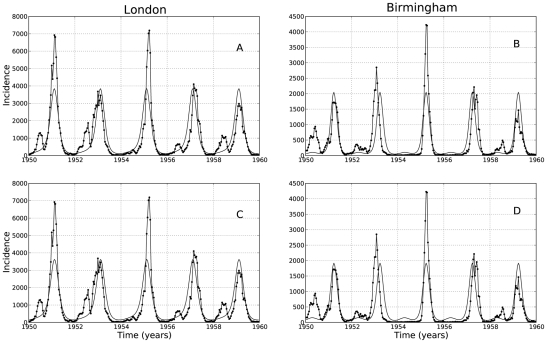
Predicted vs. observed time series of infected individuals. Using the ML estimates in Table 2, the predicted infected dynamics of the SEIR model was plotted against the number of infected individuals reported in London and Birmingham. Panels A and B show the predictions for the classical SEIR model and panels C and D show the predictions for the LHD SEIR model.

The SEIR model with classic and LHD incidence rate were fitted to measles time series data from London and Birmingham. In both cities, the SEIR-LHD model was selected as best (see Table 2). Notably, the difference in AIC and BIC is at least 2000 points in each case. The predictions for each model and city combination are shown in Figure 3. We remark that assessing and comparing the quality of the model predictions visually may be misleading. Indeed, according to our likelihood formulation, the parameter estimation process does not weight equally a deviation from the model prediction at low and high infected counts. In fact, the variance of the Poisson sampling error varies according to the mean predictions 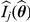.

**Table 2:** Measles SEIR model parameter estimates and model selection using a Poisson sampling model.

London									
Model	P	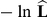	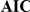	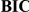	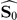	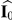	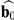	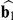	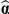
Classic	3	49881.59	99769.18	99779.85	1.4741e+05	1.1777e+02	1.5964e+03	5.0210e−02	NA
LHD	4	48102.60	96213.20	96227.43	1.5246e+05	1.5334e+02	1.5416e+03 ( 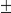 1.1340E−06)	4.8037e−02 ( 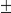 1.2461E−04)	1.5225e−05

Maximum likelihood (ML) parameter estimates for both models and two time series of the number of reported measles cases in two different cities: London and Birmingham. The sampling model for the observation error of the counts is the Poisson distribution. The letter 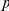 denotes the number of model parameters in each case. 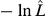 denotes the value negative log-likelihood function evaluated at the ML estimates. The AIC and BIC scores for each model vs. data set combination are also reported. The model selection decision rule is to pick the model with lowest information criterion value. Accordingly, the LHD model seems to be the best choice in both data sets. Confidence intervals for 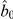 and 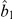 are shown in parentheses for the best model for each locality.

### Qualitative analysis of the SIRS models

In this section we discuss the differences in the qualitative behavior of the SIRS model (1)–(3) with both classical 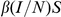 and LHD 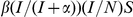 incidence rates with and without seasonal forcing. We refer the interested reader to the Text S1 for proofs of the following claims. By construction, the set 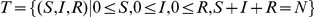 is a positively invariant set of the SIRS model (1)–(3). If we set the coefficients constant, the Dulac criterion guarantees that the SIRS model with neither the classic nor the LHD incidence rate function has periodic solutions in 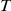. Regarding the classical incidence rate, the SIRS model has two stationary solutions: a disease free equilibrium (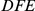) and an endemic equilibrium (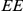). It is well known that 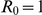 is a threshold for this model: If 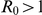 the disease remains endemic, while 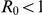 implies that the disease dies out. On the other hand, the SIRS model with LHD incidence rate has one disease free equilibrium 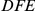 and two endemic equilibria 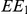 and 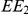. The 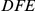 is unconditionally a local attractor. However, only one of the endemic equilibria denoted as 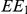, lies inside the positively invariant set 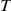. If 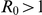 the endemic point 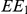 is locally an attractor. Thus, when 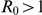 the LHD model exhibits bi-stability.

**Figure 4:**
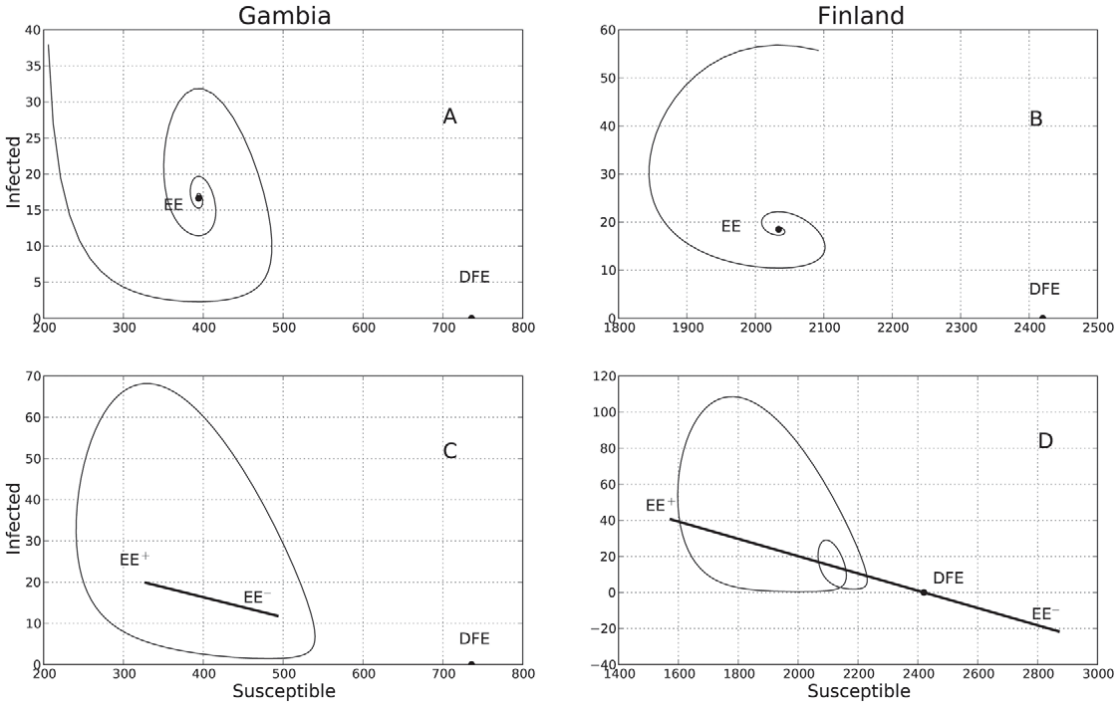
Predicted model dynamics by the classical SIRS model. Using the ML estimates in Table 1, the predicted dynamics of the classical SIRS model was plotted without seasonal forcing for both localities, Gambia and Finland (subplots A and B respectively). When seasonal forcing is added (subplots C and D), a limit cycle arises and the endemic equilibrium 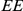 becomes a function of time (see “Qualitative analysis of the fitted SIRS models”). If the strength of seasonality 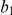 is large enough as it is the case in Finland, the limit cycle undergoes a period doubling bifurcation creating a small loop in the phase plane. This loop corresponds to the alternating small epidemic outbreaks observed in the predicted and recorded time series of infected individuals for Finland.

Introducing seasonal forcing has the following effects on the SIRS dynamics with classic incidence: first, it is well known that by letting the contact rate to be a periodic function of the form (8) where 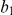 is small, the SIRS model with classical incidence rate has a periodic solution with period 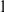. This behavior is shown in Figure 4 A and B. Also, when seasonal forcing is introduced, the basic reproductive number 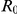 becomes a periodic function of time, 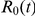, that oscillates between the values 
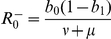
 and 
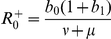
. The endemic point also becomes a periodic function of time 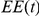 that bounces back and forth between two extreme points, 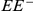 and 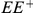. The expressions for 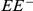 and 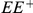 are given in the Text S1. The associated limit cycle of the model's solution inherits the stability behavior of the endemic point: if 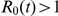, then the limit cycle is asymptotically stable. A stable limit cycle is displayed in Figure 4 C. Because the function 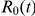 can cross the boundary of 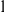 periodically depending on the value of 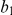, the dynamic behavior of the model's trajectory with respect to the nature of the endemic point 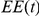 (stable/unstable) can be described with a race analogy: The model's solution can be thought of as a hopeless ‘pursuer’ engaged in a race against the endemic solution 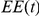 who plays the role of the fast ‘leader’ that cannot be caught upon. Just as in a cycling race, as soon as the leader changes its strategy, so does the pursuer behind the leader. In that way, if 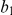 is such that 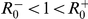 and only while 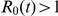, the leader (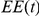) is deemed as stable and the solution's trajectory pursues the endemic point 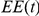. As soon as 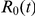 becomes less than 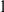, the leader ‘changes its strategy’ and is deemed unstable whereas the 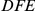 becomes stable. At that moment, the trajectory switches its objective and pursues the 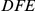 and keeps doing so while 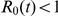. That sudden change of objective gives rise to a period doubling bifurcation of the limit cycle as seen in Figure 4 D. This change of objective (period doubling bifurcation) happens as 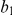 grows large. We remark that at least one route to chaos in the associated Poincaré map of this model when 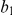 is taken as the bifurcation parameter has been shown [53], [56], [57].

**Figure 5:**
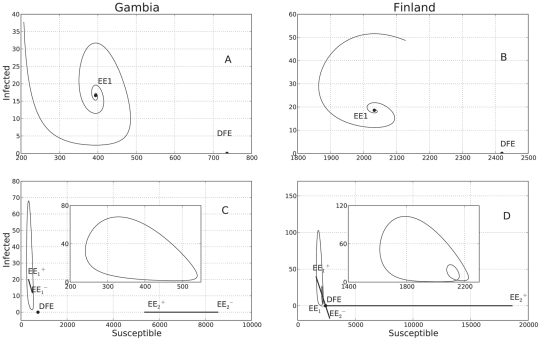
Predicted model dynamics by the nonlinear LHD SIRS model. Using the ML estimates in Table 1, the predicted dynamics of the nonlinear SIRS model with the LHD incidence rate function was plotted without seasonal forcing for both localities, Gambia and Finland (subplots A and B respectively). When seasonal forcing is added (subplots C and D), a limit cycle arises and the endemic equilibrium 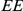 becomes a function of time (see “Qualitative analysis of the fitted SIRS models”). If the strength of seasonality 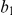 is large enough as it is the case in Finland, the limit cycle undergoes a period doubling bifurcation creating a small loop in the phase plane. This loop corresponds to the small alternating epidemic outbreaks observed in the predicted and recorded time series of infected individuals for Finland.

Finally, in the SIRS model with LHD incidence rate (see Figure 5 A and B), if we let the contact rate to be a periodic function of the form (8), a limit cycle also arises (see Figure 5 C). Here again, as 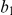 increases, the trajectory engages in the same pursuer/leader dynamics and the limit cycle undergoes a period doubling bifurcation (Figure 5 D). However, contrary to what happens in the classical SIRS model with seasonal forcing, periodicity or extinction of the epidemics depends also on the initial conditions: if the initial proportion of infected individuals is too high, the disease will die from a subsequent depletion of the susceptible pool of individuals. Only if the epidemic begins with a small number of individuals will it slowly work its way up and attain a persisting limit cycle.

## Discussion

Multiple lines of evidence show that the forced SIRS and SEIR models with LHD incidence rate function constitute a better explanation of the seasonal epidemic patterns than the corresponding classical models with seasonal forcing, for the data sets and cases explored here. The first line of evidence is statistical in nature: when confronted with different time series of seasonal epidemics, the LHD model was selected as best in three out of four cases and in the fourth case, the LHD model was nearly indistinguishable from the classic model. By formulating the fitting and the model selection problems using likelihood-based inference and information theoretic model selection criteria we were able to conclude that given the data and the models at hand our model embodies the most likely explanation of how the observed data arose. Our model's nonlinear incidence rate takes into account heterogeneity in the ability to transmit the infection while modeling the infectious process as a pure birth stochastic process and hence, it is a more realistic model formulation. This new level of model complexity was achieved by incorporating only one extra parameter. The emphasis we give to a first principles derivation that hinges on interpretability and simplicity is not always sought in other SIR-type model formulations and modeling exercises [6], [17], [18], [24], [58]. Hence, our results show that a careful exploration of other incidence rate functions before resorting to mathematically more complex, high-dimensional models may bring new insights into the current understanding of the functioning of epidemics.

Another line of evidence in favor of the LHD model comes from its qualitative predictions. The classical SIRS model without the seasonal forcing predicts somewhat artificially that regardless of the initial proportion of infected and susceptible individuals, provided 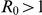, the endemic equilibrium will be reached [28]. On the other hand, the LHD model without seasonal forcing predicts that the disease-free equilibrium is always an attractor, thus exhibiting bi-stability (see qualitative analysis section). Hence, if the initial proportion of infected individuals is too high, the disease will die from a subsequent depletion of the susceptible pool of individuals, contrary to what the classical model predicts. For the disease to persist in the population, the initial proportion of infected individuals has to be very low. Only then the infection process will proceed steadily to the endemic solution. This qualitative prediction matches the virus transmission strategy that the syncytial virus seems to have evolved: recall that in our model the extra parameter 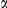 is the density of infected individuals at which the probability of successfully transmitting the infection is 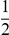. In every locality, the ML estimates of 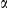 were in the order of 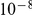 to 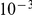, thus indicating that a very low density of infected individuals is needed in order to maximize the transmission rate of the measles and RSV diseases.

Incorporating weather covariates to our nonlinear SIRS model further improves the biological insights that can be concluded from the parameter estimation and model analysis exercises. A simple look at the strong auto-covariation patterns and at the pure weather trends, in particular for Gambia (Figure 1) indicate that modeling weather and weather effects with a sinusoidal function seems a natural add-on to the classic SIRS model, for this data set. For Gambia, the fact that the *per datum* BIC for the LHD model with the joint Poisson-Normal likelihood is very similar to the *per datum* BIC for the classic model indicates that the weather can indeed be viewed as a simple rotation and translation (eq. 15) of the weather effects (eq. 8). Thus eq. 15 may not always be viewed only as a phenomenological artifact [18]. For Finland, however, this was not the case. The *per datum* BIC favors much more clearly the Poisson likelihood formulation. Hence, we consider that in Finland the weather effects model (eq.8) would be better expressed as some unknown nonlinear transformation of the weather. In other words, in this country with more extreme weather, a change in the temperature range of a certain size is not translated as an equivalent change in the weather effects in the transmission rate. Also embedded within our weather effects model formulation (eq.8) is the hypothesis that weather affects incidence rates in a nonlinear fashion. In particular, when the strength of seasonality 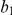 is high enough, the limit cycles predicted by both weather forced models undergo a period doubling bifurcation such that relatively small epidemic outbreaks are followed by big ones. Notably, these effects of the strength of seasonality were detected in Finland, the locality where the amplitude of the relative weather oscillation is larger.

The model selection exercise should by no means be the ending point of the analysis. Instead, if appropriateness of one model vs. the other cannot be resolved, a near-tie in a model selection situation should lead to the search and reformulation of each model's scientific predictions in a way that can be clearly tested in further experiments. Hence, the model selection results presented here should be rather viewed as the starting point of further analyses (see [59]).

Even for simple deterministic models, parameter estimation for dynamic data can be non-trivial. Dynamic models often present multimodal likelihoods thus complicating the parameter estimation process [42]. In these cases, the type of inferences possible is limited due to the presence of wide confidence sets that include parameter values with different qualitative predictions. If for instance the ML estimate of a bifurcation parameter is in a 2 limit-cycles region but its confidence interval includes parameter values for which these cycles do not appear, then there is not enough evidence in the data at hand to properly infer something about the size of the parameter of interest and hence, about the dynamic properties displayed by the data. In our case however, the precision of our parameter estimates and in particular, of the bifurcating parameter 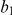 (Tables 1 and 2) is enough to identify the bifurcation region where the strength of seasonality lies for the data at hand.

Although in the two models studied here a period doubling bifurcation appears in the limit cycle, the LHD incidence rate model still provides very different qualitative predictions. In the classical model, the value of the basic reproduction number as a function of time 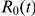 acts as a stability switch for the DFE, so that any trajectory that begins with biologically realistic initial conditions will eventually enter the limit cycle. This is not the case for the LHD model, for which the periodicity or extinction of the epidemics depends very naturally on the initial conditions. Other studies have incorporated seasonal forcing in SIRS-type models [17], [60], [61], but since all have used the classical incidence rate function, they constrain their disease persistence and epidemics predictions to whether the basic reproductive number can or cannot be periodically above 1.

**Figure 6:**
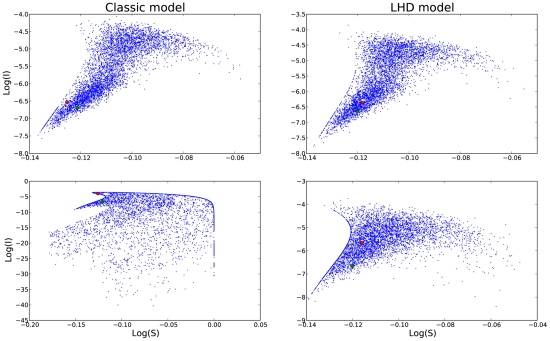
Numerical evaluation of the Poincaré map for the SIRS model with environmental stochasticity. The stationary state was initially perturbed with environmental stochasticity (green diamond). Subsequently, the map was iterated and plotted 3000 times, and at each time, environmental stochasticity was incorporated in the mapping function. The final location of the trajectory was plotted as a red diamond. In panels A and B the environmental stochasticity noise 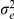 was 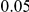 and both the initial and final states of the trajectory are in a neighborhood of the attractor. In panels C and D 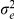 was 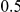. Note that in the classical model increasing the environmental noise results in transient visits to the disease free equilibrium stable submanifold (panel C), whereas in the LHD model, with a large enough perturbation the trajectory visits the disease free equilibrium basin of attraction and remains there.

Nonlinear incidence rate forms derived from first principles constitute a promising starting point to review the interaction between demographic and environmental stochasticity and nonlinear seasonal effects. Indeed, recent studies have considered including in the classic SIRS model stochasticity in the seasonal process, besides sampling and/or observation error [17]. After showing that a simple pure observation error fit of our LHD model brings about a considerable fit improvement, we explored the qualitative differences between the models by coupling the deterministic skeletons with environmental noise. In Figure 6, the depicted stochastic trajectories show that in the classical model increasing the environmental noise results in transient visits to the disease free equilibrium stable submanifold (panel c)), whereas in the LHD model, with a large enough perturbation the trajectory visits the disease free equilibrium basin of attraction and remains there. Hence, the fact that regardless of the value of the basic reproduction number the DFE is always an attractor opens the door to stochastic phenomena whereby the trajectory exits the endemic solution basin of attraction and hits just by chance the DFE basin of attraction, only when the LHD incidence rate is used. By the same token, the trajectory periodically wanders in the direction of the DFE stable submanifold (similar to the “saddle fly-by” reported by Cushing et al [54]).

The results presented here are not by any means an exhaustive exploration of the interplay between nonlinear dynamics and stochasticity, both critical factors shaping seasonal epidemic patterns. However, our results may be viewed as the starting point of multiple research avenues. Three such research topics could be: first-principles derivation of non-linear incidence rate functions, the role of bi-stability and demographic stochasticity for disease persistence and the simulation of environmental and demographic stochasticity in the Poincaré map.

## Supporting Information

Dataset S1 Dataset and Python program files. In this directory you will fine all the python code needed to reproduce the calculations in the paper, including the figures.
(2.90 MB ZIP)

Text S1 Supplementary information.
(0.21 MB PDF)
