# Supplementary material for: First Principles Modeling of Nonlinear Incidence Rates in Seasonal Epidemics
Source: PLoS Comput Biol. 2011 Feb 17;7(2):e1001079. doi: 10.1371/journal.pcbi.1001079 (PMC3040644; doi:10.1371/journal.pcbi.1001079)
Supplement: Dataset S1 — Dataset and Python program files. In this directory you will fine all the python code needed to reproduce the calculations in the paper, including the figures. (2.90 MB ZIP) [file pcbi.1001079.s001.zip › computations/README.rtf]

# Data set and python code for the data analysis # for the paper: "First principles modeling of nonlinear incidence rates in seasonal epidemics"# J.M. Ponciano and Marcos Capistran, PLoS Computational Biology 2011# Here you will find the most important python programs used in this article# The full set of programs included the code to produce the figures in this article can be found at:http://www.cimat.mx:88/~marcos/docs/plos_computations.tar.gz
