# Supplementary material for: First Principles Modeling of Nonlinear Incidence Rates in Seasonal Epidemics
Source: PLoS Comput Biol. 2011 Feb 17;7(2):e1001079. doi: 10.1371/journal.pcbi.1001079 (PMC3040644; doi:10.1371/journal.pcbi.1001079)
Supplement: Text S1 — Supplementary information. (0.21 MB PDF) [file pcbi.1001079.s002.pdf]

Supporting information for the paper: First principles modeling of nonlinear incidence rates in seasonal epidemics

José M. Ponciano[1*], Marcos A. Capistrán[2],
1 J.M. Ponciano, Department of Biology, University of Florida, Gainesville, FL 32611
2 M.A. Capistrán, Department of Mathematics, CIMAT, Guanajuato, Gto, MEXICO
∗ E-mail of Corresponding author: josemi@ufl.edu

Supporting Information

Qualitative Analysis of the SIRS Model

In this section we offer proofs of the claims made throughout section **Qualitative analysis of the SIRS models**
First, we examine the behavior of the SIRS model with LHD incidence rate. In order to exhibit that this model does not have periodic solutions in T we substite $S = N - I - R$, which leads to the system

$$\frac{dI}{dt} = \beta \frac{I}{I+\alpha}\frac{I}{N}(N - I - R) - (\nu+\mu)I = X, \tag{1}$$

$$\frac{dR}{dt} = \nu I - (\gamma+\mu)R = Y. \tag{2}$$

Now, using the Dulac function $D(I,R) = 1$, it follows that

$$\frac{\partial(DX)}{\partial I} + \frac{\partial(DY)}{\partial R} = -\frac{\beta}{N}\frac{2I^3 + 3I^2\alpha}{(I+\alpha)^2} - (\nu+\mu) - (\gamma+\mu) < 0.$$

implying that the model does not have periodic solutions in T. Regarding the stationary solutions of this model, equating the derivatives to zero

$$\mu N - \mu S - \frac{\beta}{N}\frac{I^2}{I+\alpha}S + \gamma R = 0, \quad \frac{\beta}{N}\frac{I^2}{I+\alpha}S - (\nu+\mu)I = 0, \quad \nu I - (\gamma+\mu)R = 0,$$

we obtain that there is a disease free equilibrium $(N, 0, 0)$, and two endemic equilibria given by

$$I_{1,2} = \frac{N}{2}\frac{\gamma+\mu}{\nu+\gamma+\mu}\left[\left(1 - \frac{1}{R_0}\right) \pm \sqrt{\left(1 - \frac{1}{R_0}\right) - 4\frac{\nu+\gamma+\mu}{\gamma+\mu}\frac{N\alpha}{R_0}}\right], \quad R_{1,2} = \frac{\nu}{\gamma+\mu}I, \quad S_{1,2} = \frac{N}{R_0}\frac{I+\alpha}{I}.$$

The characteristic polynomial of the linearized SIRS model with LHD incidence rate is given by

$$p(\lambda) = \begin{vmatrix} -\mu - \frac{\beta}{N}\frac{I^2}{I+\alpha} - \lambda & -\frac{\beta}{N}\frac{I^2+2I\alpha}{(I+\alpha)^2}S & \gamma \\ \frac{\beta}{N}\frac{I^2}{I+\alpha} & \frac{\beta}{N}\frac{I^2+2I\alpha}{(I+\alpha)^2}S - (\nu+\mu) - \lambda & 0 \\ 0 & \nu & -(\gamma+\mu) - \lambda \end{vmatrix} \tag{3}$$

$$= \begin{vmatrix} -\mu - \lambda & -\mu - \lambda & -\mu - \lambda \\ \frac{\beta}{N}\frac{I^2}{I+\alpha} & \frac{\beta}{N}\frac{I^2+2I\alpha}{(I+\alpha)^2}S - (\nu+\mu) - \lambda & 0 \\ 0 & \nu & -(\gamma+\mu) - \lambda \end{vmatrix} \tag{4}$$

$$= -(\mu+\lambda)\begin{vmatrix} 1 & 1 & 1 \\ \frac{\beta}{N}\frac{I^2}{I+\alpha} & \frac{\beta}{N}\frac{I^2+2I\alpha}{(I+\alpha)^2}S - (\nu+\mu) - \lambda & 0 \\ 0 & \nu & -(\gamma+\mu) - \lambda \end{vmatrix} \tag{5}$$

$$= -(\mu+\lambda)\left[\left(\frac{\beta}{N}\frac{I^2+2I\alpha}{(I+\alpha)^2}S - (\nu+\mu) - \lambda\right)(-(\gamma+\mu) - \lambda) + \frac{\beta}{N}\frac{I^2}{I+\alpha}((\nu+\gamma+\mu)+\lambda)\right] \tag{6}$$

At the disease free equilibrium the characteristic polynomial is

$$p(\lambda) = -(\mu+\lambda)((\nu+\mu)+\lambda)((\gamma+\mu)+\lambda).$$

Since all roots are negative, the disease free equilibrium is inconditionally asymptotically stable.
On the other hand, at the endemic equilibria, the caracteristic polynomial is

$$p_2(\lambda) = -(\mu+\lambda)\left(\lambda^2 + \left((\gamma+\mu) + \frac{\nu+\mu}{I_i+\alpha}\left(\frac{R_0}{N}I_i^2 - \alpha\right)\right)\lambda + \frac{\nu+\mu}{I_i+\alpha}\left(\frac{R_0}{N}I_i^2(\nu+\gamma+\mu) - (\gamma+\mu)\alpha\right)\right),$$

where $I_{1,2}$ are defined above. The stability of the endemic points is determined by the sign of the constant term $\left(\frac{R_0}{N}I_i^2(\nu+\gamma+\mu)-(\gamma+\mu)\alpha\right)$. Substituting I_i we obtain that the stability of the endemic points is determined by the sign of

$$M = \left((R_0-1) + \sqrt{(R_0-1)^2 - 4\frac{\nu+\gamma+\mu}{\gamma+\mu}\frac{R_0\alpha}{N}}\right)^2 - 4\frac{\nu+\gamma+\mu}{\gamma+\mu}1.60902719821e-06\frac{R_0\alpha}{N}, \tag{7}$$

$$N = \left((R_0-1) - \sqrt{(R_0-1)^2 - 4\frac{\nu+\gamma+\mu}{\gamma+\mu}\frac{R_0\alpha}{N}}\right)^2 - 4\frac{\nu+\gamma+\mu}{\gamma+\mu}\frac{R_0\alpha}{N}, \tag{8}$$

for $EE1$ and $EE2$ respectively.
Now, let us consider a right triangle with hypotenuse $|R_0-1|$ and catheti $\sqrt{(R_0-1)^2 - 4\frac{\nu+\gamma+\mu}{\gamma+\mu}\frac{\alpha R_0}{N}}$ and $\sqrt{4\frac{\nu+\gamma+\mu}{\gamma+\mu}\frac{\alpha R_0}{N}}$. The following statements follow immediately: Both, $M>0$ and $N<0$ if an only if $R_0>1$. Both, $M<0$ and $N>0$ if and only if $R_0<1$. Therefore, $EE1$ is asymptotically stable and $EE2$ is a saddle point if and only if $R_0>1$. Similarly, $EE1$ is a saddle point and $EE2$ is asymptotically stable if and only if $R_0<1$.

Parameter Estimates for the RSV Data Set

Table 1. RSV-SIRS model parameter estimates and model selection using a Poisson sampling model with an added weather covariate

Finland:

Model	p	$-\ln\widehat{\mathbf{L}}$	AIC	BIC	$\widehat{\mathbf{S_0}}$	$\widehat{\mathbf{I_0}}$	$\widehat{\mathbf{b_0}}$	$\widehat{\mathbf{b_1}}$	$\widehat{\alpha}$	$\widehat{\mathbf{k_w}}$
Classic	5	5174.803	10359.6100	10376.2000	2.1872E+03	88.1613	42.9507	0.2864	NA	75.01887
LHD	6	4930.835	9873.6700	9893.5780	2.2165E+03	57.58713	42.64029	0.3057	1.000E-06	43.0324

Gambia:

Model	p	$-\ln\widehat{\mathbf{L}}$	AIC	BIC	$\widehat{\mathbf{S_0}}$	$\widehat{\mathbf{I_0}}$	$\widehat{\mathbf{b_0}}$	$\widehat{\mathbf{b_1}}$	$\widehat{\alpha}$	$\widehat{\mathbf{k_w}}$
Classic	5	353.8650	717.7300	729.1133	275.6265	83.4150	67.2300	0.2020	NA	2.1535
LHD	6	353.8075	719.6150	733.2750	275.3420	28.9438	67.3154	0.2022	1.0000E-09	2.1655

Maximum likelihood (ML) parameter estimates for both models and two time series of the number of reported syncytial virus cases in two different localities: Gambia and Finland. The sampling model for the observation error of the counts is the Poisson distribution. The weather covariate observations are assumed to be normal deviates with common variance and mean given by eq. (15). The letter p denotes the number of model parameters in each case. $-\ln\widehat{L}$ denotes the value negative log-likelihood function evaluated at the ML estimates. The AIC and BIC scores for each model vs. data set combination are also reported. The model selection decision rule is to pick the model with lowest information criterion value. Accordingly, the LHD model seems to be the best choice in Finland whereas the Classical model seems to be a sufficient explanation for the observed time series patterns in Gambia.

Table 2. SIRS and SEIR model Parameters

SIRS & Weather	N	$\widehat{S}_0$	$\widehat{I}_0$	b_0	b_1	α	μ	ν	γ	c	$\bar{w}$	k_w
**Finland**												
Classic	2420	**2.187e+03**	**8.816e+01**	**4.295e+01**	**2.865e-01**	NA	0.013	36.0	1.8	**4.582e-01**	**1.404e+01**	**7.502e+01**
LHD	2420	**2.217e+03**	**8.342e+01**	**4.264e+01**	**3.058e-01**	**2.420e-03**	0.013	36.0	1.8	**4.540e-01**	**1.404e+01**	**4.303e+01**
**Gambia**												
Classic	736	**2.756e+02**	**2.898e+01**	**6.730e+01**	**2.021e-01**	NA	0.041	36.0	1.8	**2.832e-01**	**2.092e+01**	**2.164e+00**
LHD	736	**2.753e+02**	**2.894e+01**	**6.732e+01**	**2.023e-01**	**7.360e-07**	0.041	36.0	1.8	**2.832e-01**	**2.092e+01**	**2.166e+00**
**SIRS**	N	$\widehat{S}_0$	$\widehat{I}_0$	b_0	b_1	α	μ	ν	γ	c	$\bar{w}$	k_w
**Finland**												
Classic	2420	**2.195e+03**	**8.681e+01**	**4.285e+01**	**2.914e-01**	NA	0.013	36.0	1.8	**4.633e-01**	NA	NA
LHD	2420	**2.186e+03**	**9.496e+01**	**4.288e+01**	**2.708e-01**	**5.883e-03**	0.013	36.0	1.8	**5.078e-01**	NA	NA
**Gambia**												
Classic	736	**2.756e+02**	**2.898e+01**	**6.730e+01**	**2.021e-01**	NA	0.041	36.0	1.8	**2.833e-01**	NA	NA
LHD	736	**2.756e+02**	**2.897e+01**	**6.730e+01**	**2.021e-01**	**7.360e-07**	0.041	36.0	1.8	**2.832e-01**	NA	NA
**SEIR**	N	$\widehat{S}_0$	$\widehat{I}_0$	b_0	b_1	α	μ	ν	σ	c	k_w	$\bar{w}$
**London**												
Classic	3249440	**1.474e+05**	**1.178e+02**	**1.596e+03**	**5.021e-02**	NA	0.02	73.0	45.625	**1.900e-01**	NA	NA
LHD	3249440	**1.525e+05**	**1.533e+02**	**1.542e+03**	**4.804e-02**	**1.523e-05**	0.02	73.0	45.625	**1.943e-01**	NA	NA
**Birmingham**												
Classic	1106465	**5.812e+04**	**6.229e+01**	**1.331e+03**	**1.503e-01**	NA	0.02	73.0	45.625	**2.309e-01**	NA	NA
LHD	1106465	**6.214e+04**	**9.171e+01**	**1.254e+03**	**1.456e-01**	**1.574e-08**	0.02	73.0	45.625	**2.411e-01**	NA	NA

The estimated parameters are in boldface.
